# Semi-extensive management system drives lower prevalence of equine orodental disorders

**DOI:** 10.3389/fvets.2026.1827412

**Published:** 2026-08-18

**Authors:** João Ricardo Kunz, Rubens Peres Mendes, Milena Carol Sbrussi Granella, Bettina Hartl, Anderson Fernando de Souza, Joandes Henrique Fonteque, Silvio Kau-Strebinger

**Affiliations:** 1 Departamento de Medicina Veterinária, Centro de Ciências Agroveterinárias, Universidade do Estado de Santa Catarina, Lages, Brazil; 2 Departamento de Cirurgia, Faculdade de Medicina Veterinária e Zootecnia, Universidade de São Paulo, São Paulo, Brazil; 3 Unit of Morphology, Department of Biological Sciences and Pathobiology, University of Veterinary Medicine Vienna, Vienna, Austria

**Keywords:** breeding system, dental health, dentistry, feeding, horse, husbandry

## Abstract

**Introduction:**

The prevalence of orodental disorders in horses kept under different management systems is lacking. More detailed knowledge is needed to support targeted therapeutic and prophylactic approaches, as these conditions play a crucial role in maintaining equine health and welfare. This study aimed to compare the prevalence of orodental disorders in horses raised, kept, and fed under extensive, semi-extensive, or intensive management systems.

**Methods:**

Sixty-nine clinically healthy adult crossbred horses with no history of dental treatment were examined, with 23 horses included in each study group previously maintained under three different management systems. All horses underwent a clinical evaluation, and an orodental examination was performed under standing sedation.

**Results:**

Incisors (*p* = 0.013), premolar and molar diastemata (*p* < 0.001), as well as irregular incisors (*p* = 0.001), occurred more frequently in horses kept under extensive systems. Horses in the intensive system showed a higher prevalence of oral mucosal ulcers (*p* = 0.001) and excessive transverse ridges in premolars and molars (*p* = 0.016). Only dorsal curvature malocclusion was more common in horses managed under semi-extensive systems (*p* = 0.018). No differences were observed in the overall number of orodental alterations among the systems.

**Conclusion:**

We concluded that management systems are associated with the frequency and severity of dental abnormalities, with the semi-extensive system showing a lower incidence and severity than the others.

## 1 Introduction

Dental abnormalities are commonly observed in horses due to morphological characteristics and their peculiar feeding habits (1). The hypsodont dentition of horses results in continuous tooth eruption to compensate for occlusal wear, including abrasion and attrition that occur during the mastication of fiber-rich feeds (1–4). In their natural environment, horses graze for approximately 18 h per day. However, many horses are managed under restricted conditions with limited or no access to grazing, receiving carbohydrate-rich diets in fractioned feedings (2, 5).

Diet has an important influence on the development of periodontal disease (6). Studies suggest that dental alterations, particularly caries, are more likely to occur in horses fed excessive amounts of carbohydrate (7, 8). Additionally, diets containing moderate amounts of concentrated feed have been associated with concomitant dental conditions (9).

The frequency of dental changes in horses kept under different management systems is scarcely discussed in the literature. Western Australian horses maintained on pasture for at least 8 months were less likely to develop peripheral caries than those with limited pasture access (8). A study conducted on working donkeys in Mexico, maintained under different feeding conditions, highlighted the influence of diet type and feeding management on dental wear and the occurrence of oral lesions (10). On the other hand, it is believed that horses managed intensively may develop more dental abnormalities due to decreased lateral excursion during mastication, depending on the feed type and the reduced chewing time (1, 2, 4).

The aim of this study was to determine the prevalence of dental disorders in horses maintained under extensive, semi-extensive, and intensive management systems and with no history of dental treatment. We hypothesized that extensive management would promote better oral health, resulting in a lower prevalence of orodental disorders.

## 2 Methods

### 2.1 Animals

This observational cross-sectional study included a total of 69 client-owned adult horses of different breeds, both male and female, that had been previously maintained under extensive, semi-extensive, or intensive management systems. Animals were not experimentally allocated to management conditions; rather, they were selected according to their pre-existing husbandry system at the time of evaluation. Horses underwent a general clinical examination, and any abnormality identified during this assessment was considered an exclusion criterion. Only horses with no history of prior dental treatment were included, and animals that had previously undergone dental procedures or oral cavity evaluation were excluded. Horses managed extensively had a mean age of 17.2 ± 6.0 years (range: 9–31 years), semi-extensively managed horses had a mean age of 15.4 ± 3.9 years (range: 5–21 years), and intensively managed horses had a mean age of 13.0 ± 5.2 years (range: 8–20 years). Age was determined from historical records in 46 horses and by evaluation of the occlusal surface of the incisor teeth in 23 horses.

### 2.2 Study design

The horses were divided into three groups (*n* = 23 each) according to their management system: extensive, semi-extensive, or intensive. The horses kept under an extensive grazing system came from a single property, where they were fed exclusively (year-round) on native pasture in the spring and summer (September through March) and on a mixture of oat and ryegrass pasture in the fall and winter (April through August), with unlimited access to pasture. The horses raised under a semi-extensive system were partially stabled, receiving alfalfa hay and commercial feed mixed with ground corn, and were allowed to graze on native pastures for approximately 12 h per day. The horses raised under an intensive system came from a single property, were fully confined, received alfalfa hay three times a day and commercial feed twice a day, and were only taken out of their stalls for three to 4 h of work. All horses were fed at ground level.

After collecting all relevant information about the animals, the horses underwent a specific clinical dental examination. The procedures were performed with the horses at rest, under sedation. For the dental evaluation, the horses were sedated with detomidine hydrochloride at a dose of 0.02–0.04 mg/kg, administered intravenously.

### 2.3 Oral cavity examination

All horses first underwent a general clinical examination and then a detailed orodental assessment as described by Easley and Tremaine (11). Access to the oral cavity was achieved using an oral speculum (Conrad Speculum, AAA Equine Equipment Inc., NJ, USA) using a dental mirror and a headlight. All examinations were performed by a single experienced clinician (JRK). Inspection and external palpation of the head and soft tissues, palpation of the temporomandibular joint (TMJ), and intraoral evaluation were performed to identify dental abnormalities. Dental overgrowths were identified and localized using the modified Triadan system, and findings were recorded on individual dental charts.

For the evaluation of the incisors, the presence or absence of anomalies was assessed, such as occlusal irregularities, excessive diagonal bites, and ventral and dorsal curvatures, as well as diastemata. Diagonal bites (DGL) were assessed using a specific 2-dimensional clinical orthodontic angle measuring device. The instrument consists of 2 elongated bars, 1 transverse cross bar (for measuring DGL), connected to a graduated scale (DGL: −20° to +20°) (12). Following Pellachin (13), any deviation greater than one degree was considered positive for a DGL. They were then classified according to the specific quadrants exhibiting overgrowth. Overgrowth involving the quadrants 100 and 300 was classified as DGL 3, whereas involvement of the quadrants 200 and 400 was classified as DGL 4 (14).

For the assessment of the cheek teeth and associated soft tissues, the following parameters were recorded: the presence of rostral and caudal hooks, ramps, excessive transverse ridges (ETR), excessive sharp enamel points (ESEPs), mucosal ulcerations, steps, waves, infundibular cemental alterations (IC), peripheral dental caries (PDC), diastemata, and dental fractures. In this study, the local probability of occurrence of diastemata was estimated independently of periodontal disease status. Consistent with previous studies (15, 16), clinical staging of periodontitis, periodontal pocket depth, and classification of diastemata as open or closed were not considered. All widened interdental spaces (IDS) were defined as diastemata, irrespective of feed accumulation, gingival recession, or periodontal pocketing.

Infundibular cemental alterations and peripheral dental caries were defined according to previous description, using the modified Honma Classification system (17). Small central defects within the occlusal infundibular cementum were considered normal residual vascular channels associated with the blood supply of the developing infundibulum. Brownish-black discoloration affecting the occlusal infundibular or peri-infundibular dental hard tissues, with or without associated larger occlusal cemental hypoplasia, was classified as IC. Demineralization and carious discoloration affecting peripheral dental hard tissues were classified as PDC. Teeth were classified only according to the presence or absence of these alterations, without severity grading.

Secondary sagittal dental fractures resulting from carious coalescence of the mesial and distal infundibula were included in the dental fracture group. Dental fractures were recorded as a single category, irrespective of whether they involved enamel or pulp, and were not further subclassified according to anatomical extent or severity.

### 2.4 Data analysis

Statistical analyses were performed using SigmaPlot 14.5. Associations between management systems and orodental disorders were evaluated using the Chi-square test. Normality of data distribution was assessed using the Shapiro–Wilk test. Comparisons of the overall number of orodental disorders among management systems were performed using One-way Analysis of Variance (ANOVA). Differences were considered statistically significant when *p* < 0.05.

## 3 Results

**Figure 1:**
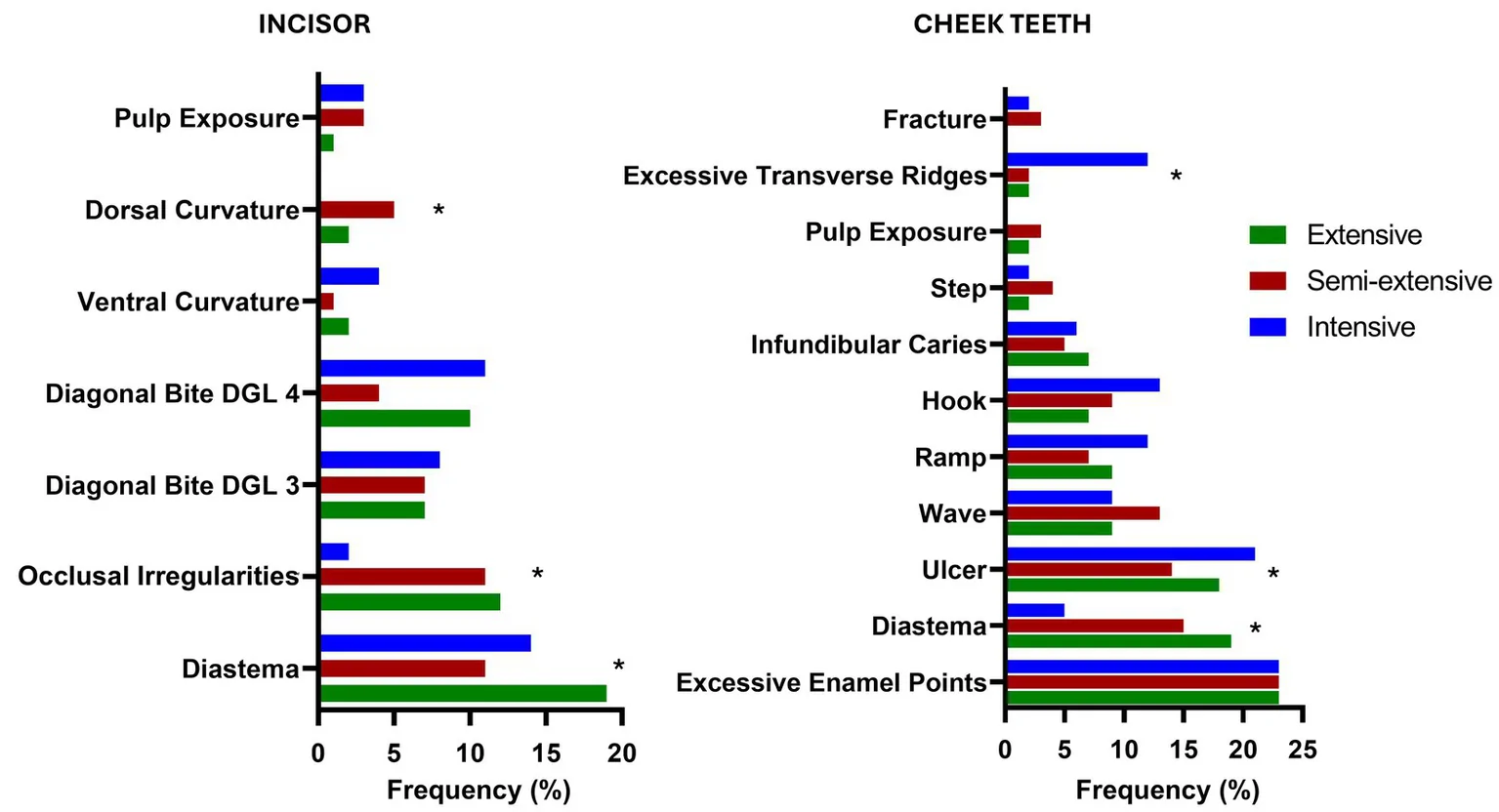
Number of horses affected by incisor and cheek tooth (premolar and molar) orodental disorders in 69 adult horses managed under extensive (*n* = 23), semi-extensive (*n* = 23), and intensive (*n* = 23) systems. ^*^Chi-square test; *p* < 0.05 indicates a significant effect of management system. DGL3, diagonal bite with overgrowth of quadrants 100/300; DGL4, diagonal bite with overgrowth of quadrants 200/400.

Among incisor disorders, diastemata represented the most frequent abnormality and differed significantly among management systems (*χ*^2^ = 6.15, *p* = 0.046), occurring more frequently in horses maintained under extensive management. Occlusal irregularities also differed among groups (*χ*^2^ = 11.42, *p* = 0.003), being more frequently observed in extensive and semi-extensive systems and less commonly detected under intensive management. Dorsal curvature was significantly associated with management system (*χ*^2^ = 6.04, *p* = 0.049), occurring most frequently in horses managed semi-extensively. In contrast, DGL abnormalities (DGL3 and DGL4), ventral curvature, and pulp exposure showed similar distributions across management systems (Figure 1; Table 1).

**Table 1:** Number and frequency (%) of incisor and cheek tooth disorders in horses maintained under different management systems.

Disorder	Management system			*p*-value^a^
	Extensive (%)	Semi-extensive (%)	Intensive (%)	
Diastema (PM/M)	19 (82.6)	15 (65.2)	5 (21.7)	<0.001
Diastema (I)	19 (82.6)	11 (47.8)	14 (60.7)	0.046
Soft tissues (ulcers) (PM/M)	18 (78.3)	14 (60.9)	21 (91.3)	0.049
Occlusal irregularities (I)	12 (52.2)	11 (47.8)	2 (8.7)	0.003
Dorsal curvature (I)	2 (8.7)	5 (21.7)	0 (0)	0.049
ETR (PM/M)	2 (8.7)	2 (8.7)	12 (52.2)	<0.001

a Differences among groups were evaluated using the chi-square test. I, incisors; PM/M, premolars and/or molars; ETR, excessive transverse ridges.

Regarding cheek tooth disorders, sharp enamel points were observed in all horses (69/69), regardless of management system, and were therefore excluded from comparative analyses. Premolar and molar diastemata represented the most frequent cheek tooth abnormality and showed marked differences among management systems (*χ*^2^ = 18.40, *p* < 0.001), occurring more frequently in horses maintained under extensive and semi-extensive systems. Oral mucosal ulcerations (*χ*^2^ = 6.02, *p* = 0.049) and excessive transverse ridges (*χ*^2^ = 16.27, *p* < 0.001) also differed significantly among management systems and were more commonly observed in intensively managed horses. Wave mouth, hooks, and ramps showed numerically higher frequencies in intensive systems (*p* > 0.05). Cheek tooth fractures were identified in 5 of 69 horses (7.2%), occurring only in the semi-extensive and intensive groups, with no differences among management systems (*p* > 0.05). Likewise, pulp exposure and step mouth occurred at low frequency across all groups (Figure 1; Table 1).

The mean number of orodental disorders per animal did not differ among management systems (extensive: 3.78 ± 1.31; semi-extensive: 4.09 ± 1.28; intensive: 4.22 ± 1.41; *p* = 0.529) (Table 2). Despite the similar overall presence of orodental disorders among groups, horses maintained under extensive management exhibited a numerically higher frequency of several specific dental abnormalities (Table 1; Figure 1).

**Table 2:** Mean number of orodental disorders per horse according to management system (extensive, semi-extensive and intensive).

Management system	Mean	Standard deviation	*p*-value^a^
Extensive	3.78	1.31	0.529
Semiextensive	4.09	1.28	
Intensive	4.22	1.41	

a Comparison using one-way ANOVA, where *p* < 0.05 indicates a significant effect of management systems.

## 4 Discussion

This study aimed to determine the prevalence and distribution of orodental disorders in horses maintained under three distinct management systems. The findings allowed a partial acceptance of the initial hypothesis that extensive management would favor better oral health. Contrary to expectations, horses kept under extensive conditions showed a higher frequency of several specific dental abnormalities, whereas semi-extensive management was associated with comparatively fewer and less severe disorders. This represents the central outcome of the study and highlights the influence of management conditions on the pattern of orodental disorders observed. Overall, the results suggest that management systems combining pasture access with controlled feeding may be associated with more favorable outcomes for specific oral health parameters influenced by management, whereas other conditions with genetic or developmental origins are likely less affected by management practices.

The higher occurrence of incisor abnormalities in horses maintained under extensive systems, particularly diastemata and irregular incisors, may be related to longer chewing times and the more rigid structure of fibrous diets, which increase the likelihood of interdental food impaction and occlusal imbalance. Although horses in the extensive group had a slightly higher mean age, this difference alone does not support a direct association between the observed abnormalities and age-related changes. Instead, these alterations are likely multifactorial and may reflect a combination of acquired occlusal changes, progressive dental wear, and dietary influences (18). Horses managed extensively obtain food directly through grazing and therefore rely more heavily on their incisors for forage apprehension, whereas animals maintained under intensive systems have been shown to preferentially use their lips for food intake (19). Such differences in feeding behavior may contribute to the greater occurrence of incisor abnormalities observed under extensive management.

A similar rationale may explain the occurrence of incisor curvature abnormalities, particularly dorsal curvature, which appears to be influenced by feeding behavior and incisor use during forage apprehension. The prevalence of incisor curvatures was similar to that found in a population of Crioulo horses from Brazil and Chile (20, 21). In contrast, ventral curvature may be more closely associated with developmental factors, particularly differences in the timing of eruption between upper and lower central incisors.

Diagonal incisors have consistently been reported at low frequencies in previous studies, ranging from 0.9 to 11.8% [Salem et al. (22), 0.9%; Vemming et al. (23), 7.5%; Muñoz et al. (21), 9.8%; and Leite et al. (20), 11.8%]. In contrast, DGL were identified in 48 of 69 horses (69.6%) in the present study, representing one of the most frequently observed incisor abnormalities. This discrepancy is likely related to methodological differences, particularly the use of a dedicated measuring device that allows accurate detection and classification of DGL, including subtle deviations that may be overlooked during visual inspection alone. Additionally, in some previous studies horses were not sedated and no specific measuring instrument was employed (20, 22), which may have contributed to underestimation of this condition.

Sharp enamel points were among the most prevalent conditions observed across all management systems, occurring in nearly all horses evaluated, regardless of feeding regimen. Similar findings have been consistently reported in the literature, with sharp enamel points described in horses managed under both intensive and extensive conditions (4) as well as in Brazilian cart horses (15), slaughter horses (23), and large clinical populations (24). Although the occurrence of sharp enamel points has been associated with feeding habits (1), their high prevalence supports the concept that they represent a common morphological feature in horses. However, the clinical relevance of these abnormalities likely depends on severity, as more pronounced enamel points may promote trauma to the oral mucosa and ulcer formation (2). In this context, the greater occurrence of oral mucosal ulcers in intensively managed horses may reflect increased severity of enamel point formation, potentially associated with reduced lateral mandibular excursion during mastication of concentrate-based diets (1, 2).

Diastemata represented one of the most relevant findings in the present study, particularly in horses maintained under extensive management. This pattern may be associated with longer chewing times and the more rigid structure of fibrous diets, which increase the likelihood of interdental food impaction and progressive occlusal wear. Similar observations have been reported in previous studies, which also associated pasture-based diets with increased interdental spacing (24). In addition, the absence of prior dental procedures in all horses evaluated may have contributed to the persistence of occlusal irregularities predisposing to secondary diastema formation. Because no peripheral caries were identified in the evaluated population, the observed diastemata are more likely associated with mechanical alterations in occlusal wear and feeding behavior than with caries-related processes.

The higher occurrence of infundibular caries in horses with unrestricted access to pasture contrasts with the observations of Dixon and Du Toit (25) but is consistent with findings reported by Simhofer et al. (24) and Salem et al. (22). More recent evidence suggests that the pathogenesis of infundibular caries may be more strongly influenced by intrinsic developmental factors, particularly infundibular cementum hypoplasia, which predisposes to food retention, bacterial colonization, and progression of carious lesions (26, 27). Therefore, the higher prevalence observed in extensively managed horses may not be exclusively attributable to management conditions, but rather to individual development and possibly genetic factors. This interpretation is supported by Fitzgibbon et al. (16), who identified infundibular cementum hypoplasia and/or dental caries in a high proportion of horses examined using computed tomography.

Wave mouth was most frequently observed in horses managed semi-extensively. Similar results were found by Vemming et al. (23) and contrasted with those observed by Salem et al. (22). The etiology of this condition is not fully understood, but wave formations are thought to develop when multiple teeth in a sequence become elongated relative to their opposing teeth. Such elongation may result from delayed eruption of permanent teeth, for example due to retention of deciduous teeth, with premature loss of the affected teeth being a common consequence (2, 5).

According to Dixon and du Toit (25), step mouth may develop following tooth loss or fractures, leading to excessive overgrowth of the opposing tooth due to reduced occlusal wear. In the present study, however, the number of steps observed exceeded the number of fractures identified. Moreover, the fractures detected in premolars and molars were limited to partial fractures of the clinical crown and showed no apparent association with step formation. Therefore, it is likely that the observed steps developed as a consequence of delayed eruption of permanent teeth or differences in density between opposing teeth, resulting in asymmetric wear, or due to temporal differences in deciduous tooth exfoliation.

Cheek tooth fractures were uncommon and showed no apparent association with management system. Comparable findings have been reported previously by Dixon et al. (28), Leite et al. (20), and Salem et al. (22). Although idiopathic fractures involving the pulp cavity or infundibulum may predispose affected teeth to apical infection (29), the fractures identified in the present population were limited to the clinical crown and were not associated with overt clinical signs.

Hooks and ramps were observed at a higher frequency in horses managed under intensive systems compared with the other groups. Similar findings were reported by Leite et al. (20), who observed this condition in 46% of horses older than 5 years. These occlusal overgrowths may impair performance by causing soft tissue trauma resulting from pressure exerted by the bit against the affected teeth (30). Pagliosa et al. (19) reported that feeding grains and concentrates shortens the masticatory cycle, thereby promoting the formation of rostral and caudal hooks. In addition, feeding at elevated levels restricts rostrocaudal mandibular movement, contributing to the development of ramps and hooks (31). However, in the present study, all horses were fed at ground level, ruling out above-ground feeding as a contributing factor to the higher frequency of hooks and ramps observed.

Regarding the presence of excessive transverse ridges (ETR), the differences observed in the present study corroborate the findings of O’Neill et al. (4), who reported a prevalence of 45% in stabled horses compared with 4% in horses raised extensively. Likewise, Leite et al. (20) observed a prevalence of 21.3% of ETR in Crioulo horses maintained under extensive conditions. These findings reinforce the association between ETR, feeding practices, and management conditions. Concentrate-based diets with low abrasive properties may reduce lateral mandibular excursion and impair occlusal wear efficiency, potentially contributing to uneven wear between opposing dental arches due to differences in enamel distribution across occlusal surfaces (1).

This study has some limitations. The cross-sectional design does not allow assessment of temporal progression or causal relationships between management conditions and the development of orodental disorders. Also, age distribution differed slightly among groups, which may have influenced the expression of certain dental abnormalities, despite all animals having no history of dental treatment. Future studies may benefit from stratifying horses by age within management systems to minimize potential age-related confounding. In addition, the absence of oroscopic examination represents a limitation, as this is more sensitive than conventional mirror inspection for detecting subtle oral alterations (24, 32). Radiographic assessment was not performed in cases of tooth fractures or diastemata; therefore, the full clinical significance and extent of these conditions could not be determined. Furthermore, environmental factors, feeding history, and individual variation in dental eruption patterns could not be fully controlled. Future longitudinal studies including larger populations and comprehensive imaging approaches may help clarify the mechanisms underlying the associations observed.

## 5 Conclusion

Semi-extensive management systems were associated with fewer and less severe orodental disorders in horses compared with extensive and intensive conditions. Different management systems may be associated with the incidence and severity of orodental disorders, however, there is no evidence to suggest that specific management systems are associated with specific types of lesions.

## Data Availability

The raw data supporting the conclusions of this article will be made available by the authors, without undue reservation.

## References

[1] Bonin SJ Clayton HM Lanovaz JL Johnston T. Comparison of mandibular motion in horses chewing hay and pellets. Equine Vet J. (2007) 39:258–62. doi: 10.2746/042516407x157792, 17520978

[2] Dixon PM. Removal of equine dental overgrowths. Equine Vet Educ. (2000) 12:68–81. doi: 10.1111/j.2042-3292.2000.tb01768.x

[3] Dixon PM Dacre I. A review of equine dental disorders. Vet J. (2005) 169:165–87. doi: 10.1016/j.tvjl.2004.03.022, 15727909

[4] O’Neill HVM Keen J Dumbell L. A comparison of the occurrence of common dental abnormalities in stabled and free-grazing horses. Animal. (2010) 4:1697–701. doi: 10.1017/S1751731110000893, 22445123

[5] Johnson T. J. Porter C. M. Dental overgrowths and acquired displacement of cheek teeth. Proceedings of the annual convention of the American Association of equine practitioners. (2006). 1–4.

[6] Dacre I. "The impact of nutrition on dental health and management of equine teeth for optimal nutrition". In: Applied Equine Nutrition. (ed.) Lindner A. Wageningen: Brill (2005). p. 27–41.

[7] Borkent D Dixon PM. Equine peripheral and infundibular dental caries: a review and proposals for their investigation. Equine Vet Educ. (2017) 29:621–8. doi: 10.1111/eve.12497

[8] Jackson K Kelty E Tennant M. Equine peripheral dental caries: an epidemiological survey assessing prevalence and possible risk factors in Western Australian horses. Equine Vet J. (2018) 50:79–84. doi: 10.1111/evj.12718, 28707363

[9] Borkent D Reardon RJM McLachlan G Smith S Dixon PM. An epidemiological survey on the prevalence of equine peripheral dental caries in the United Kingdom and possible risk factors for its development. Equine Vet J. (2017) 49:480–5. doi: 10.1111/evj.12610, 27423159

[10] du Toit N Burden FA Dixon PM. Clinical dental findings in 203 working donkeys in Mexico. Vet J. (2008) 178:380–6. doi: 10.1016/j.tvjl.2008.09.013, 18977674

[11] Easley J Tremaine WH. "Dental and oral examination". In: Equine Dentistry. (eds). Easley J Dixon PM Schumacher J. Saint Louis: W.B. Saunders (2011). p. 185–98.

[12] Kau S Motter KS Moser VJ Kunz JR Pellachin M Hartl B . Intra- and interexaminer measurement variability analysis of an orthodontic gauge device to determine incisor occlusal surface angles in the horse. Vet Sci. (2022) 9:481. doi: 10.3390/vetsci9090481, 36136698 PMC9506125

[13] Pellachin M. Objective measurements of occlusal angles. Proceedings of the 11th IGFP conference Wiesbaden Germany. (2013). 45–57

[14] DeLorey MS. A retrospective evaluation of 204 diagonal incisor malocclusion corrections in the horse. J Vet Dent. (2007) 24:145–9. doi: 10.1177/089875640702400302, 17985690

[15] Kunz JR Granella MCS Mendes RP Müller TR Kau S Fonteque JH. High prevalence of orodental disorders in south Brazilian cart horses: walking a tightrope between animal welfare and socioeconomic inevitability. J Vet Dent. (2020) 37:149–58. doi: 10.1177/0898756420968306, 33118460

[16] Fitzgibbon CM Du Toit N Dixon PM. Anatomical studies of maxillary cheek teeth infundibula in clinically normal horses. Equine Vet J. (2010) 42:37–43. doi: 10.2746/042516409X474761, 20121911

[17] Honma K Yamakawa M Yamauchi S Hosoya S. Statistical study on the occurrence of dental caries in domestic animals: I. Horse. Jpn J Vet Res. (1962) 10:31–6. doi: 10.14943/jjvr.10.1.31

[18] Ireland JL McGowan CM Clegg PD Chandler KJ Pinchbeck GL. A survey of health care and disease in geriatric horses aged 30 years or older. Vet J. (2012) 192:57–64. doi: 10.1016/j.tvjl.2011.03.021, 21550271

[19] Pagliosa GM Alves GES Faleiros RR Saliba EOS Sampaio IBM Gomes TLS . Influência das pontas excessivas de esmalte dentário na digestibilidade e nutrientes de dietas de eqüinos. Arq Bras Med Vet Zootec. (2006) 58:94–8. doi: 10.1590/S0102-09352006000100014

[20] Leite CT Duarte CA Mozzaquatro FD Mistieri MLA Machado IRL Porciuncula ML . Levantamento de afecções dentárias em equinos da raça Crioula mantidos em sistema de criação extensivo. Arq Bras Med Vet Zootec. (2019) 71:21–7. doi: 10.1590/1678-4162-10331

[21] Muñoz L Vidal F Sepúlveda O Ortiz O Rehhof C. Patologías dentales en incisivos, caninos y primer premolar en caballos chilenos adultos. Arch Med Vet. (2010) 42:85–90. doi: 10.4067/S0301-732X2010000100012

[22] Salem SE Townsend NB Refaai W Gomaa M Archer DC. Prevalence of oro-dental pathology in a working horse population in Egypt and its relation to equine health. Equine Vet J. (2017) 49:26–33. doi: 10.1111/evj.12533, 26526823

[23] Vemming DC Steenkamp G Carstens A Olorunju SAS Stroehle RM Page PC. Prevalence of dental disorders in an abattoir population of horses in South Africa by oral examination of intact and bisected heads. Vet J. (2015) 205:110–2. doi: 10.1016/j.tvjl.2015.03.021, 25979819

[24] Simhofer H Griss R Zetner K. The use of oral endoscopy for detection of cheek teeth abnormalities in 300 horses. Vet J. (2008) 178:396–404. doi: 10.1016/j.tvjl.2008.09.029, 19041805

[25] Dixon PM Du Toit N. "Dental anatomy". In: Equine Dentistry. (eds). Easley J Dixon PM Schumacher J. Saint Louis: W.B. Saunders (2011). p. 51–76.

[26] Suske A Pöschke A Schrock P Kirschner S Brockmann M Staszyk C. Infundibula of equine maxillary cheek teeth. Part 1: development, blood supply and infundibular cementogenesis. Vet J. (2016) 209:57–65. doi: 10.1016/j.tvjl.2015.07.029, 26832811

[27] Suske A Pöschke A Müller P Wöber S Staszyk C. Infundibula of equine maxillary cheek teeth: part 2: morphological variations and pathological changes. Vet J. (2016) 209:66–73. doi: 10.1016/j.tvjl.2015.11.023, 26831172

[28] Dixon PM Tremaine WH Pickles K Kuhns L Hawe C Mccann J . Equine dental disease part 3: a long-term study of 400 cases: disorders of wear, traumatic damage and idiopathic fractures, tumours and miscellaneous disorders of the cheek teeth. Equine Vet J. (2000) 32:9–18. doi: 10.2746/042516400777612099, 10661379

[29] Dacre I Kempsot S Dixon PM. Equine idiopathic cheek teeth fractures. Part 1: pathological studies on 35 fractured cheek teeth. Equine Vet J. (2007) 39:310–8. doi: 10.2746/042516407x182721, 17722721

[30] Tell A Egenvall A Lundström T Wattle O. The prevalence of oral ulceration in Swedish horses when ridden with bit and bridle and when unridden. Vet J. (2008) 178:405–10. doi: 10.1016/j.tvjl.2008.09.020, 19027332

[31] Lima JTM Andrade BSC Schwarzbach SV De Marval CA Leal BB Faleiros RR . Ocorrência de doença infundibular, sobremordida e ganchos em equinos de cavalaria militar. Arq Bras Med Vet Zootec. (2011) 63:6–11. doi: 10.1590/S0102-09352011000100002

[32] Chiero NE Reiswig JD Griffin CE Panigrahi KJ Gardner AK. Blinded comparison of mirror and endoscopic oral examination in the horse: sensitivity, specificity and observer agreement. Equine Vet Educ. (2023) 35:e227–33. doi: 10.1111/eve.13698

